# As We Drink and Breathe: Adverse Health Effects of Microcystins and Other Harmful Algal Bloom Toxins in the Liver, Gut, Lungs and Beyond

**DOI:** 10.3390/life12030418

**Published:** 2022-03-14

**Authors:** Apurva Lad, Joshua D. Breidenbach, Robin C. Su, Jordan Murray, Rebecca Kuang, Alison Mascarenhas, John Najjar, Shivani Patel, Prajwal Hegde, Mirella Youssef, Jason Breuler, Andrew L. Kleinhenz, Andrew P. Ault, Judy A. Westrick, Nikolai N. Modyanov, David J. Kennedy, Steven T. Haller

**Affiliations:** 1 College of Medicine and Life Science, University of Toledo, Toledo, OH 43614, USA; apurva.lad@rockets.utoledo.edu (A.L.); joshua.breidenbach@rockets.utoledo.edu (J.D.B.); robin.su@rockets.utoledo.edu (R.C.S.); jordan.murray@rockets.utoledo.edu (J.M.); rebecca.kuang@rockets.utoledo.edu (R.K.); alison.mascarenhas@rockets.utoledo.edu (A.M.); john.najjar@rockets.utoledo.edu (J.N.); shivani.patel8@rockets.utoledo.edu (S.P.); prajwal.hegde@rockets.utoledo.edu (P.H.); mirella.youssef@osumc.edu (M.Y.); jason.breuler@rockets.utoledo.edu (J.B.); andrew.kleinhenz@utoledo.edu (A.L.K.); nikolai.modyanov@utoledo.edu (N.N.M.); 2 Department of Chemistry, University of Michigan, Ann Arbor, MI 48109, USA; aulta@umich.edu; 3 Lumigen Instrumentation Center, Department of Chemistry, Wayne State University, Detroit, MI 48202, USA; judy.westrick@wayne.edu

**Keywords:** harmful algal blooms, cyanotoxins, microcystin-LR, pre-existing disease

## Abstract

Freshwater harmful algal blooms (HABs) are increasing in number and severity worldwide. These HABs are chiefly composed of one or more species of cyanobacteria, also known as blue-green algae, such as *Microcystis* and *Anabaena*. Numerous HAB cyanobacterial species produce toxins (e.g., microcystin and anatoxin—collectively referred to as HAB toxins) that disrupt ecosystems, impact water and air quality, and deter recreation because they are harmful to both human and animal health. Exposure to these toxins can occur through ingestion, inhalation, or skin contact. Acute health effects of HAB toxins have been well documented and include symptoms such as nausea, vomiting, abdominal pain and diarrhea, headache, fever, and skin rashes. While these adverse effects typically increase with amount, duration, and frequency of exposure, susceptibility to HAB toxins may also be increased by the presence of comorbidities. The emerging science on potential long-term or chronic effects of HAB toxins with a particular emphasis on microcystins, especially in vulnerable populations such as those with pre-existing liver or gastrointestinal disease, is summarized herein. This review suggests additional research is needed to define at-risk populations who may be helped by preventative measures. Furthermore, studies are required to develop a mechanistic understanding of chronic, low-dose exposure to HAB toxins so that appropriate preventative, diagnostic, and therapeutic strategies can be created in a targeted fashion.

## 1. Harmful Algal Blooms and Cyanotoxins

Harmful algal blooms (HABs) develop when colonies of algae grow out of control and harm marine life, humans, and terrestrial animals [1]. Algae is a broad classification including single and multicellular plant life as well as dinoflagellates and autotrophic bacteria. However, HABs are often chiefly composed of one or more species of cyanobacteria, also known as blue-green algae, such as *Microcystis* and *Anabaena*, and can form dense scum-like layers in both marine and freshwater bodies [2,3] The frequency and intensity of these blooms worldwide have risen significantly in recent decades. Some of the most notable water bodies affected by these cyanobacterial blooms are Lake Winnipeg, Canada; Lake Erie, USA; Lake Victoria, Kenya; and Lake Taihu, China [4]. A rapidly warming climate and increased eutrophication of water bodies due to anthropogenic activities are the main causes of HABs. These events have severe consequences for ecological systems at all levels and damage the socio-economic status of the surrounding regions [5].

One of the significant factors contributing to the rampant growth of HABs is the eutrophication of water reservoirs. Run-off water enriched in nitrogen and phosphorus from agricultural and industrial processes nourishes the organisms, including *Microcystis* species, which is one of the most dominant bloom-forming and toxin-producing cyanobacterial species [6]. In a study conducted by Krausfeldt et al., the researchers noted that differences in the source of nitrogen or nitrogen isotopes grossly influenced the metabolism and toxin composition of *Microcystis aeruginosa.* They observed that cells grown in urea as a nitrogen source produced the most toxic congener of the toxin microcystin [7]. Chaffin et al. determined that both *Microcystis* and *Planktothrix* can use nitrate, ammonium, or organic nitrogen (i.e., urea) as nitrogen sources and that high light intensities enhance microcystin toxin production during periods of elevated nitrogen concentrations [8]. This highlights the importance of considering nitrogen as well as phosphorus in developing strategies to mitigate cyanobacterial blooms.

Another critical but often overlooked aspect that significantly contributes to the growth of cyanobacterial blooms is zooplankton grazing. In a recent study conducted by Ladds et al., the authors studied nutrient enrichment and zooplankton grazing combined with next generation sequencing and fluorometric analyses to quantify their effects on specific cyanobacterial genera across the western basin of Lake Erie. The authors found that Daphnid (a type of crustaceous plankton) grazing significantly reduced the net growth of *Planktothrix* in Sandusky Bay as well as *Dolichospermum* net growth in the Maumee Bay areas of Lake Erie, USA, both of which are bloom-forming cyanobacterial species. In contrast, the toxin-producing *Microcystis* species was unaffected by the plankton grazing and mainly depended on nutrient enrichment [9]. This study established that plankton grazing can effectively reduce certain species of bloom-forming cyanobacteria while nutrient limiting strategies can eliminate others such as *Microcystis*.

Of the vast number of species of cyanobacteria found around the world, the most pervasive bloom-forming cyanobacterial species belong to the genus *Microcystis*. During winter, these organisms are benthic, overwintering, and rise to the surface to form blooms during favorable conditions [10,11,12]. Many species of *Microcystis* are known to produce secondary metabolites termed cyanotoxins. These secondary metabolites are often toxic to higher trophic organisms and pose an increased environmental risk to human and animal health. These toxic metabolites are classified into hepatotoxins, neurotoxins, cytotoxins, and dermatoxins [3]. For a more thorough review of these metabolites, Jones et al. describes the creation of an extensive and comprehensive database named “CyanoMetDB” [13]. This open access database is a comprehensive collection of 2010 cyanobacterial metabolites as well as 99 structurally related compounds curated from 850 peer-reviewed articles published between 1967 and 2020. This database provides an extensive and detailed insight into the occurrence, functions, mechanisms, and toxicological risks posed to humans and animals from these secondary metabolites. 

Importantly, some cyanobacterial strains exert toxic effects despite not producing any known cyanotoxins, thus indicating the presence of potentially unknown or uncharacterized toxins. In fact, there are numerous experimental works that show the neurotoxic, hepatotoxic, and cytotoxic action of cyanobacterial extracts with no known cyanotoxins [14,15,16,17], underscoring the need to discover and characterize potential new toxins and/or bioactive compounds produced by HABs. In interesting research conducted by Spoof et al., the researchers have isolated and identified new bioactive, cyclic hexapeptides—Anabaenopeptins—from a cyanobacterial bloom extract in the Baltic Sea and found the compounds to inhibit the activity of protein phosphatase 1 and carboxypeptidase A but no inhibition of chymotrypsin, trypsin, or thrombin [18]. In another research performed by Bittner et al., the researchers have shown that complex mixtures of cyanobacterial extracts containing bio-active compounds other than the secondary metabolites can exert cytotoxic as well as genotoxic effects in the form of DNA strand breaks in in vitro (human hepatocarcinoma cell line HepG2 cells) conditions [19]. 

**Figure 1:**
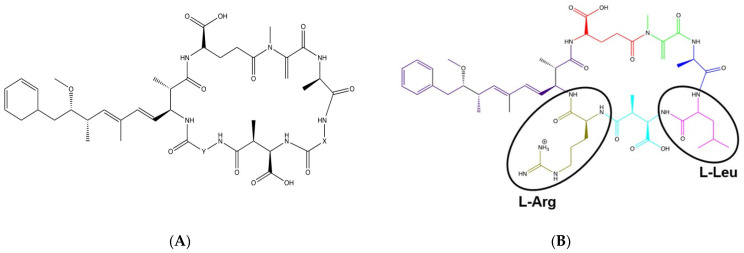
Structure of Microcystin and Microcystin-LR. (**A**) General structure of Microcystin with X and Y as variable amino acids at positions 2 and 4, respectively; (**B**) Structure of Microcystin-LR where L stands for L-Leucine and R represents L-Arginine.

Of the variety of toxins produced, Microcystis are well known to produce microcystins (MCs), a class of hepatotoxins. The toxicokinetics, molecular toxicology, and pathophysiology of microcystins has been recently and comprehensively reviewed by Arman and Clarke [20]. There are over 300 different congeners of microcystins identified to date [21]. MCs are cyclic heptapeptides with two conventional amino acids in positions X and Y and a unique β-amino acid ADDA (3-Amino-9-methoxy-2,6,8-trimethyl-10-phenyldeca-4,6-dienoic acid) (Figure 1A) [22]. The two conventional amino acids are variable, which contributes to the various congeners of the toxin [10]. These different congeners vary in toxicity based on hydrophobicity and their ability to form a chemical bond between the toxin and the protein phosphatases within cells such as hepatocytes, which can subsequently damage the cells [23].

**Figure 2:**
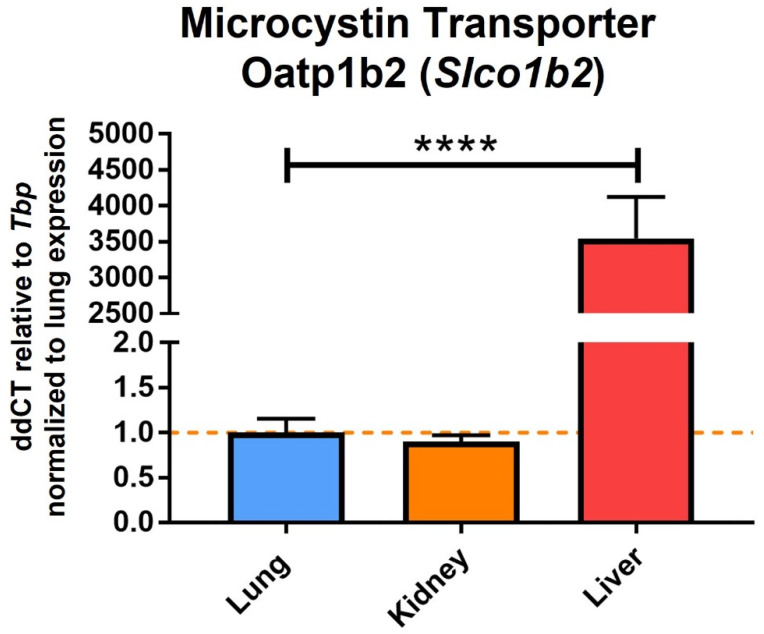
Genetic expression of microcystin transporter Oatp1b2 (*Slco1b2*) in different organs of C57Bl/6J male mice. Real-time PCR analysis of microcystin transporter Oatp1b2-*Slco1b2* in lung, kidney, and liver of healthy C57Bl/6J male mice was conducted as previously reported [31]. The mice were 15 weeks old at the time of harvest and the organs were flash frozen in liquid nitrogen upon harvest. All values are relative to housekeeping gene TATA-binding protein (*Tbp*) and normalized to expression levels in lung tissue. Statistical analysis by Student’s *t*-test (*n* = 3, **** = *p* ≤ 0.0001).

One of the most common and potently toxic congeners of microcystin is Microcystin-LR (MC-LR), which contains amino acids leucine (L) and arginine (R) in the X and Y positions, respectively (Figure 1B). MC-LR is a chemically stable compound, and the routes of exposure to this toxin include inhalation, ingestion, or dermal contact with contaminated waters [24]. Once inside, the toxin is taken up by the cells through the organic anion transporting polypeptides (OATPs) and specifically inhibits the serine/threonine protein phosphatases (PP)-PP1 and PP2A. This results in the hyperphosphorylation of proteins leading to alterations in the cytoskeleton with subsequent disruption of the cells, including cell lysis. MC-LR is also known to increase oxidative stress triggering apoptosis and pyroptosis processes as well as promoting tumor progression [3,25,26]. Although exposure to MC-LR can affect all organ systems, studies have shown that the liver is a major target organ due to the expression of the microcystin transporting and uptake channels including Oatp1b2 [27], Oatp1b1, and Oatp1b3 [28,29]. In fact, our lab has found that in C57BL/6J mice, the liver expresses Oatp1b2 (*Slco1b2*) several orders of magnitude higher compared to organs such as the lung and kidney (Figure 2, unpublished work). The International Agency for Research on Cancer (IARC) has classified MC-LR as a Group 2B peptide, i.e., the agent (peptide) is a possible carcinogen to humans [30]. Studies per-formed in rats and mice demonstrated tumor promotion mechanisms and development of preneoplastic lesions in liver and colon on exposure to sub-chronic levels of MC-LR which was attributed to the inhibition of PP1 and PP2A. Studies indicate that these toxins modulate genetic expression of early response genes, oncogenes, and markers of inflammation, all of which affect cell division, cell survival, and apoptosis.

## 2. Adverse Health Effects of HAB Toxins across Major Organ Systems

While there have been many cases of cyanotoxin-induced injuries, two early large-scale incidents of human injury and death resulting from cyanotoxin exposure have been well-documented and studied in detail over the years. The first case was a major outbreak of hepatoenteritis in Palm Island, Australia in 1979 where a total of 140 children and 10 adults were hospitalized for symptoms including malaise, anorexia, vomiting, headache, bloody diarrhea, dehydration, and painful hepatomegaly [32,33]. These patients also demonstrated acute kidney disease and liver failure with elevated serum enzyme levels. This outbreak was directly linked to Solomon Dam, a potable water reservoir which was contaminated with toxic cyanobacterium *Cylindrospermopsis raciborskii*. The bloom was treated with copper sulphate, a common algaecide, that led to cyanobacterial cell lysis and subsequent massive release of the toxin cylindrospermopsin (CYN) into the water supply. This incident highlighted the adverse effects of exposure to CYN in humans and the need to strategize bloom management techniques to neutralize the toxins from the water. The second incident was cyanotoxin exposure that occurred at a hemodialysis center in Caruaru, Brazil, in 1996 where the lack of reverse osmosis in the filtration system led to contamination of the water with MCs and CYN, which was used to prepare the dialysate. In this incident 116 of the 131 patients receiving treatment developed headache, blurred vision, eye pain, nausea, and vomiting. Of these, 100 patients developed acute liver failure and more than half of them died [34]. More recently, HAB events across the US have been responsible for human illnesses and animal deaths [35], as well as a “Do not drink” state of emergency for the residents of Northwest Ohio in 2014 [36]. The World Health Organization (WHO) established guidelines on permissible limits of microcystin exposure in water based on studies performed in healthy animals. In one of the key toxicologic studies supporting these guidelines, mice were exposed to varying doses of MC-LR, and a No Observable Adverse Effect Level (NOAEL) was established to be 40 μg/kg of body weight per day, and a Low Observable Adverse Effect Level (LOAEL) was established to be 200 μg/kg of body weight per day for liver pathology [37]. After appropriately calculating for interspecies variation, the permissible limit for safe exposure was set as 1 μg/L in humans [37,38,39,40]. Although a significant amount of research has been done to study the toxic effects of MC-LR on liver, not much is known about the adverse effects of the toxin in the setting of common pre-existing diseases such as liver or gastrointestinal disease.

While there is ample literature regarding the effects of HAB toxins such as MCs in healthy model systems, it is important to examine the ways that pre-existing chronic illness may modify susceptibility to toxicity as well. Recent surveillance from the United States Centers for Disease and Control’s *One Health Harmful Algal Bloom System* (spanning 2016–2018) confirm that exposure to HABs occurs primarily in public, outdoor recreation areas during the summer months [41]. Long-term exposure to MCs and other toxins may occur in people that frequently work or recreate in affected bodies of water, inhale aerosolized toxins, consume diets composed of large quantities of seafood that live in HAB-affected water, or ingest supplements contaminated with cyanotoxins [42,43,44].

The health effects of HAB toxins such as MCs are not limited to one particular organ system. For instance, while MCs may be typically classified as a hepatotoxin, their effects extend beyond the liver. For instance, chronic (8 month) exposure to HAB toxins such as MC-LR also have harmful effects on the renal system including necrosis, hyalinization of renal interstitium, hemorrhages, and infiltration of leukocytes, as shown by Milutinovic et al. in Wistar rat models [45]. Overall impaired function of the kidneys has been correlated with MC and aflatoxin exposure in humans [46]. In the gastrointestinal system, colorectal/rectal carcinomas in humans may be worsened by microcystin exposure [47]. Gastrointestinal cells in the duodenum, ileum, and jejunum may display increased apoptosis, resulting in gastroenteritis [48,49]. A study was done using a human genome program and asthmatic bronchial epithelium transcripts for analysis. Specific genes involved in asthma development and progression were identified, and an analysis showed that they were involved in a response to toxic substances which were targeted by plant products and plant-related toxins. Among these toxins was microcystin, showing that those with asthma may have increased susceptibility to microcystin [50]. As for the cardiovascular system, exposure to microcystins has also resulted in pathophysiological changes such as declines in stroke volume, cardiac output, heart rate/blood pressure, and oxygen consumption. This may translate to diseases such as cardiac arrest, hypovolemic shock, and hypotension in humans [51,52]. The final system we explored was the effects of HAB exposure on neurological disorders. For neurodegenerative disorders, other HAB toxins such as β-Methylamino-L-alanine or BMAA, in addition to microcystins, are important to take into account due to their interactions which have been proposed to enhance neurotoxicity. There is evidence to suggest that exposure to cyanotoxins is a major risk factor for Amyotrophic Lateral Sclerosis (ALS), a condition that impacts the nervous system and limits physical function [53]. Some studies have even reported that brain tissue taken from patients who died of Alzheimer’s Disease, amyotrophic lateral sclerosis, or Parkinson’s Disease in Guam and Canada showed elevated levels of BMAA compared to brains of those who died from other causes [54]. While a number of excellent reviews have provided a comprehensive review of affected organ systems [20,55], we focus on summarizing the literature related to the intersection of MCs’ effect on liver, gut, and pulmonary organ systems with an emphasis on how pre-existing diseases of these organ systems may increase susceptibility.

**Figure 3:**
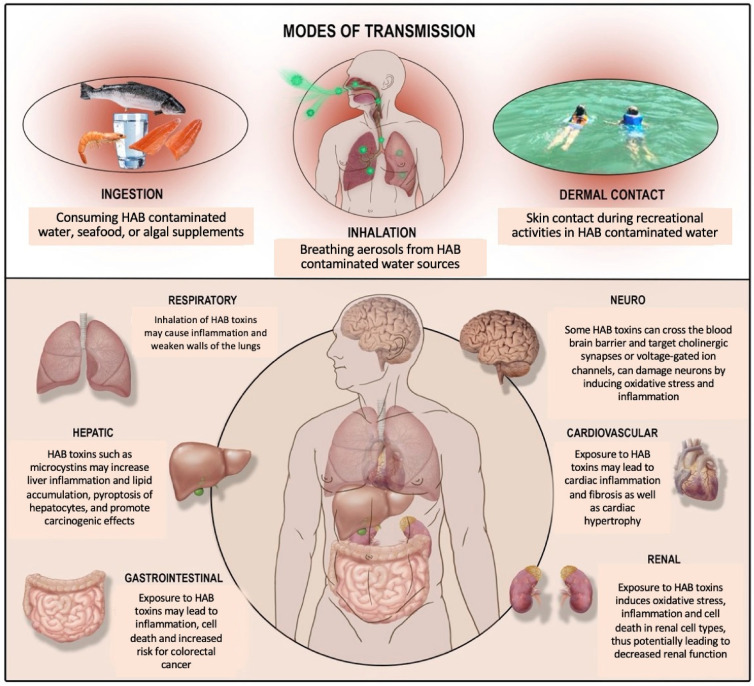
Visual representation of routes of exposure to cyanotoxins in humans and the different affected organ systems. Data referenced from [2,57,58,59].

Additionally, the U.S. Environmental Protection Agency Office of Water, Health and Ecological Criteria Division has provided an exceptional support document comprehensively detailing the health effects of MCs as summarized from both experimental animal models and observational epidemiologic data [56]. Figure 3 provides an overview of how HAB toxins may be transmitted into humans and highlights affected organ systems.

## 3. Microcystins and Liver Health

Liver diseases are the 12th leading cause of death in the United States [60], and given the known hepatic effects of MCs, patients with pre-existing liver diseases represent a potentially vulnerable population. Non-alcoholic Fatty Liver Disease (NAFLD) is a spectrum of liver conditions defined by the presence of steatosis in more than 5% of the hepatocytes with little to no alcohol consumption [61]. This condition is more prevalent in obese and diabetic individuals but is also present in lean, non-diabetic people and is considered a metabolic syndrome [61,62,63]. NAFLD is rapidly increasing in the United States as well as in other developed countries. NAFLD is a benign condition with excess fat accumulation in the liver. The more severe form of NAFLD is called as Non-alcoholic Steatohepatitis (NASH). NASH is a progressive form of NAFLD and is characterized by hepatocellular ballooning, steatosis, inflammation, and fibrosis. In an attempt to regenerate the lost liver tissue, NASH can progress to cirrhosis leading to an increase in the formation of scar tissue. Cirrhosis is an end stage organ failure that can lead to liver carcinoma and may require liver transplantation [61]. According to Fazel et al., around 27–34% of the general population throughout North American is affected by NAFLD. This condition affects almost 75–92% of obese individuals and around 60–70% of diabetic patients [64]. Although diabetes and obesity are the primary risk factors promoting NAFLD condition, factors such as ethnicity, body weight, body-mass index, sex, and exposure to environmental toxins also play a role in NAFLD onset and progression [65,66].

Some studies have demonstrated a potential link between MC-LR exposure and its contribution to the development of NAFLD pathogenesis. In 2015, Zhang et al. performed a unique study correlating incidences of algal blooms and the prevalence of NAFLD cases in the United States using a satellite imaging technique [67]. Other studies have shown that intraperitoneal or oral exposure to low doses of MC-LR altered hepatic lipid content, increased steatosis and markers of oxidative stress such as catalase and superoxide dismutase, as well as glutathione content in the plasma of exposed rodents [39,40,66,68].

Although results from all these studies suggest that cyanotoxin exposure may lead to the development and progression towards NAFLD, they do not consider the effect of the MC exposure when liver diseases such as NAFLD are *pre-existing*. This is an important distinction, because of the impact that diseases such as NAFLD and NASH have on xenobiotic metabolism. For instance, it is well-known that the presence of NASH alters hepatic drug transporter mechanisms. In a study performed by Canet et al. [69], the authors investigated diet-induced as well as genetic models of NASH in both rats and mice and observed the induction of efflux transporters and repression of uptake transporters in both liver mRNA and protein expression analyses. Due to the similarity of the human transporter mRNA and protein expression to the rodent models, their data suggested that these rodent models can aptly be used to infer similar altered drug metabolism and toxicokinetics in humans.

To bridge the gap in knowledge regarding MC-LR toxicity in the presence of pre-existing NAFLD, we used a genetic murine model of NAFLD to mimic the various aspects of NAFLD in human populations [31]. In this study, we observed that chronic low dose oral exposure to MC-LR even at levels 2.5–4.5 times lower than the acceptable limits lowered their survival rate and increased hepatic injury via excess fat accumulation in the hepatocytes. It was also observed that exposure to low levels of the toxin upregulated genes associated with hepatotoxicity, cholestasis and oxidative stress as well as affected the phosphorylation pattern of proteins that are involved in inflammation, immune response cycle and development. It should also be noted that there were significant differences observed in the toxin excretion levels of healthy mice as compared to the NAFLD mice indicating differential biodistribution and metabolism in the NAFLD mice, where healthy mice excreted nearly 60 times more MC in their urine compared to NAFLD mice.

Similarly, two studies performed by Clarke et al. lend support to these findings [66,70]. In one study, the authors demonstrate that presence of high fat/high cholesterol diet-induced NAFLD in rats altered the toxicokinetics of acute MC-LR toxicity by causing the rats to have increased hepatic inflammation, plasma cholesterol, proteinuria, and renal injury after a single acute dose of MC-LR given via intravenous or intraperitoneal injection [66]. In another study, the authors performed a sub chronic exposure to MC-LR in Sprague Dawley rats that were fed with either normal, methionine and choline-deficient (MCD) or high fat/high cholesterol (HFHC) diet for 10 weeks. After 6 weeks on the diet, the rats were injected intraperitoneally with either 0, 10, or 30 μg/kg of MC-LR every 48 h for 4 weeks. The authors observed increased inflammation and fibrosis in the liver as well as alteration in the expression of genes involved in de novo lipogenesis and fatty acid esterification in the rats exposed to MC-LR as compared to the control group indicating that MC-LR toxicity in the context of pre-existing NASH may drive the liver to a more severe phenotype that resembles end-stage NASH [70].

Chronic exposure to HAB toxins such as MC-LR may also exacerbate liver cancer and is associated with a cellular phenotype that includes hepatocyte necrosis, cell blebbing, and cell fragmentation [71]. As mentioned above, prolonged low-dose exposure to MC-LR has also been associated with a NASH phenotype, potentially setting the stage for hepatocellular carcinomas [72]. In an in vitro study done by Diez-Quijada et al., the authors investigated the adverse effects of a combination of algal toxins [cylindrospermopsin (CYN) and MC-LR] in HepG2 cells [73]. The combination induced DNA double-strand breaks after 72 h exposure, while cell cycle analysis revealed that CYN and CYN/MCLR arrested HepG2 cells in G0/G1 phase. Moreover, they also observed upregulation of *CYP1A1* and target genes involved in DNA-damage response (*CDKN1A*, *GADD45A*). On the other hand, MC-LR (1 μg/mL) alone did not have any effect on either cell viability or cell division. The results from this study underscore the importance of not only examining HAB toxins by themselves, but also in the various combinations that are sure to exist in nature. In studies performed by Arman et al., rats with diet-induced NASH that were exposed to sub-chronic levels of MC-LR showed increased fibrosis and inflammation compared to the control group [70]. These rats were then put on a 4-week recovery period after MC-LR exposure, and they observed that mice with pre-existing NASH continued to show dysregulation of the genes related to cellular differentiation and hepatocellular carcinoma [74]. This indicates that exposure to HAB toxins in pre-existing liver disease not only impairs hepatic recovery but that carcinogenic effects may also persist even after withdrawal from the exposure.

To date, clinicians around the world do not have any means to determine definite exposure to cyanotoxins and must resort to a diagnosis of exclusion as the symptoms of potential cyanotoxin exposure often overlap those of other illnesses. Therefore, there is a need to establish diagnostic methods that will aid in the differential diagnosis of potential HAB exposure. Our group has investigated the suitability of common clinical markers of liver injury, specifically alanine aminotransferase (ALT) and alkaline phosphatase (ALP), as potential diagnostic tools for liver damage induced by chronic low dose administration of MC-LR in the setting of pre-existing NAFLD [75]. We found that while MC-LR induced significant histopathologically confirmed liver damage in the setting of NAFLD, both gene expression and serum levels of ALT and ALP failed to increase with MC-LR exposure. In a human HepG2 liver epithelial cells model which has been used to simulate NAFLD in vitro [76], we observed that increasing MC-LR exposure did not lead to an increase in ALT or ALP gene expression, intracellular enzyme activity, or extracellular activity, despite a significant increase in MC-LR-induced cytotoxicity. These findings suggest that common liver injury markers such as ALT and ALP may be unsuitable as diagnostic biomarkers for chronic or low-dose MC-LR-induced liver damage.

## 4. Microcystins and Gut Health

While much of the literature on MC-LR is focused on liver toxicity, there is a paucity of knowledge on its effects on other organ systems such as the gastrointestinal (GI) tract. Indeed, the gut is the first site of MC-LR absorption following ingestion and studies have shown that the GI tract is the location with the highest bioaccumulation of the toxin [40,77,78,79]. Evidence from experimental models has shown that MC-LR is primarily absorbed by the small intestine, induces intestinal apoptosis, ROS generation, and promotes intestinal inflammation [40,48,80]. In a study by Botha et al. [48], the authors investigated the apoptotic effect of intraperitoneal MC-LR administration on the GI tract including duodenum, jejunum and ileum of mice. They noted that the apoptotic index was significantly raised in all portions of the small intestine 8 h after exposure and continued to rise even 32 h after exposure. The duodenum showed the most significant increase in apoptotic index, followed by the jejunum and ileum. Immunohistochemistry analysis indicated the presence of MC-LR in the lamina propria, suggesting a role for MC-LR in the induction of apoptosis in the GI tract of mice exposed to a single sublethal dose of MC-LR. In another study by Lun et al., the authors investigated the association between MCs in drinking water and colorectal cancer in humans [47]. The study spanned over a period of 19 years, collecting 408 cases of colon and rectal carcinoma. The authors noted that the incidence of colorectal cancer was significantly higher in the population of patients who obtained drinking water from sources with greater MC concentrations (e.g., rivers and ponds) vs. those who drank from uncontaminated sources (e.g., well water or treated tap water). Thus, there is evidence to suggest that HAB toxin exposure may adversely affect the GI tract.

To elucidate MC-LR toxicity in the gut in regard to pre-existing disease setting, our group has examined one of the most prevalent GI-related disorders, inflammatory bowel disease (IBD), as a model of pre-existing GI disease. IBD is an umbrella term, which includes ulcerative colitis and Crohn’s disease, to describe conditions involving chronic inflammation of the GI tract. IBD is the result of various genetic and environmental factors and is one of the most common GI diseases around the world, affecting around 3.1 million individuals in the US alone [81]. Many of the environmental factors exacerbating IBD include smoking, diet, various medications, and microbial infections such as *H. pylori*, *M. avium*, and *E. coli* [82].

To study the effect of MC-LR in pre-existing IBD settings, we chose a dextran sulfate sodium (DSS)-induced colitis murine model. Exposure to DSS resulted in weight loss, splenomegaly, and severe colitis marked by transmural acute inflammation, ulceration, shortened colon length, and bloody stools [57]. On further exposure to MC-LR, the mice experienced prolonged weight loss and bloody stools, increased ulceration of colonic mucosa, and shorter colon length as compared with DSS mice. Exposure to MC-LR also resulted in greater increases in pro-inflammatory transcripts within colonic tissue (TNF-α, IL-1β, CD40, MCP-1) and the pro-fibrotic marker, PAI-1, as compared to DSS-only ingestion [57]. Mice with pre-existing colitis that were exposed to the toxin showed significantly higher macrophage infiltration in the colonic tissues as compared to the non-exposed mice [83]. Interestingly, the pro-inflammatory mediator CD40 was significantly upregulated on exposure to MC-LR, a molecular feature shared with IBD progression [84]. Therefore, to identify the mechanism of MC-LR toxicity in the gut, we demonstrated that a murine CD40 knock-out model attenuates the effects of MC-LR in mice with pre-existing colitis by decreasing the severity of weight loss, allowing a full recovery in bloody stools, preventing the exacerbation of colonic shortening, preventing the exacerbation of colonic ulceration, and preventing the upregulation of the pro-inflammatory and pro-fibrotic cytokines [85]. Similar effects were also demonstrated by administration of a CD40 receptor blocking peptide that ameliorated the effects of MC-LR exposure [85]. These findings demonstrate that exposure to MC-LR exacerbates the severity of pre-existing colitis in a CD40 dependent manner and that targeting this pathway therapeutically may be conceptually attractive.

MCs’ impact on the gut is not confined to mammalian species. In another study done in *Lithobates catesbeiana* (American bullfrog) tadpoles, it was observed that acute, short-term exposure of tadpoles to HAB toxins containing 1 μg/L (1 nmol/L) of total microcystins for only 7 days resulted in significant liver and GI toxicity [86]. MC-LR-exposed tadpoles showed increased intestinal diameter, decreased intestinal fold heights, and a constant number of intestinal folds, indicating pathological intestinal distension, similar to toxic megacolon. HAB-toxin-exposed tadpoles also demonstrated hepatocyte hypertrophy with increased hepatocyte binucleation consistent with carcinogenic and oxidative processes within the liver. Both livers and intestines of HAB-toxin-exposed tadpoles demonstrated significant increases in protein carbonylation consistent with oxidative stress and damage. These findings underscore the need to evaluate the GI-related effects related to HAB toxin exposure, including MCs. This also highlights the need to evaluate the influence HAB toxins may have on other vulnerable species within the food web and how those may ultimately also impact human health [86].

## 5. Microcystins and Pulmonary Health

While exposure to cyanotoxins is typically investigated after ingestion routes of exposure, MC-LR has recently been detected in aerosols generated from HAB water [87], suggesting that it may be aerosolized by bubble bursting, wave crashing, and recreational activity in the HAB-affected water [88,89,90]. While there are survey reports and case studies of detectable concentrations of microcystins in airway mucosa as well as clinically significant airway irritation in subjects who have been near HAB-containing bodies of water for short periods [91,92], we are only aware of one study that has modeled MC-LR aerosol inhalation exposure [93]. In this study, nose-only inhalation exposure to 260-265 μg/m^3^ for 0.5, 1, or 2 h each day for 7 days resulted in minimal to moderate multifocal degeneration and necrosis along with neutrophilic inflammation only in the nasal cavity. However, the investigators were unable to confirm the delivery of the aerosol to the central or peripheral airways. In another thorough investigation, MC-LR was delivered by intratracheal injection to mice at various concentrations resulting in a lethal dose (75 μg/kg) and cause of death (liver hemorrhaging) seemingly identical to intraperitoneal injection [94]. In a study conducted by Oliveira et al., 6–7-week-old male Swiss mice were exposed via intranasal instillation of 10 μL of 6.7 ng/kg MC-LR or vehicle distilled water control once a day for 30 days and significant increases in granulocytes were found in histological analysis of the lung tissue [95]. Similarly, in other studies in which exposure was systemic, the effects on lungs were primarily granulocytic inflammation [96,97]. Picanco et al. conducted a study in which whole cyanobacterial extracts were administered by intraperitoneal injection to 4-week-old and 12-week-old mice resulting in significant increases in pulmonary granulocytic inflammation after 2 days in both groups [96]. In a follow-up study, this same group reported that 6–8-week-old male Swiss mice which were administered 40 μg/kg of specifically MC-LR by intraperitoneal injection, demonstrated significant increases in granulocytes found in histological analysis of whole lung tissue at time points of 2, 8, 24, 48, and 96 h after injection [97]. Since these exposures had the common response of granulocytic inflammation in the pulmonary tissue, even in the context of a systemic exposure, this suggests that an aerosol exposure may have similar effects. Therefore, further investigations may be warranted to determine the threat to potentially at-risk individuals with pre-existing airway inflammatory disease.While there are survey reports and case studies of airway irritation in subjects who have been near affected bodies of water for short periods, there are no thorough investigations of lung function in recreational or occupational settings or those living near the affected bodies of water [91,98]. Furthermore, these aerosols can travel over 30 km from the affected source, thus exposing wide segments of the population to both inhalation and dermal exposure [99]. These points are underscored by the fact that while the average human drinks ~2 L of fluid per day, the same average person respires/processes over 11,000 L of air per day. This fact alone draws attention to the importance of understanding how cyanotoxins affects the airways and major organ systems of individuals exposed to its aerosol in order to develop rational and targeted strategies to deal with these exposures, especially in vulnerable and at-risk populations.

## 6. New Insights into Microcystin Detection

The public health implications of HAB events have drawn some serious attention and necessitated the development of highly sensitive, reliable, and selective analytical tools for detection and identification of HAB toxins. Many studies have been performed to detect cyanotoxins such as MC-LR in blood, tissues, urine, and feces. Commonly used methods of detection include enzyme-linked immunosorbent assays (ELISA) [100,101,102], High-performance liquid chromatography (HPLC) combined with ultraviolet [103] or mass spectrometric detection [104,105,106], protein phosphatase inhibition assay (PPIA) [107,108,109], and capillary electrophoresis (CE) [110,111]. Liquid chromatography with tandem mass spectrometry (LC-MS-MS), however, is a powerful detection tool with the added advantage of being able to separate and identify various MC congeners and can be used for accurate and precise quantification of MCs in blood, urine and tissues [112,113]. In fact, we have developed a robust Solid Phase Extraction method to extract MCs from biofluids (e.g., plasma and urine) and tissue (e.g., liver) and combined with LC-MS technique to further identify the different congeners of MC as well as improve the sensitivity in order to detect low levels of the toxin. The sensitivity and reproducibility were able to be improved using a UHPLC-triple quadrupole (QqQ)-MS/MS method [112,113,114].

To study the biodistribution of the toxin in various tissues, we utilized matrix-assisted laser desorption/ionization (MALDI) imaging in order to study toxin distribution in solid organs such as the liver [115]. Both covalently bound and free MC-LR can be found in the liver of mice exposed to the toxin, and our results indicate that the distribution of free microcystins in tissue sections from affected organs, such as the liver, can be monitored with high-resolution MALDI-MS imaging [115]. These techniques give us a three-dimensional view of the distribution of those cyanotoxins in different organs and provide biologically relevant insights regarding the distribution and metabolism of these potent and harmful cyanotoxins.

## 7. Corrective Measures

Over the years, various strategies have been developed and implemented to control the spread of algal/cyanobacterial blooms [6,116]. First and foremost, there is a need to control the nutrient input of nitrogen and phosphorus into the water reservoirs. Other physical controls include aeration or mechanical mixing of the water causing the cyanobacteria to move in the vertical direction as well as limiting nutrient accessibility. Sonication, i.e., using ultrasound technology to rupture the cyanobacterial cells is an effective method for smaller water bodies, whereas the surface skimming method is used for clearing blooms in later stages. Chemical methods include the use of algaecide or barley straw to inhibit the growth of cyanobacteria. Flocculation and coagulation are two other methods that facilitate sedimentation of nutrients and cyanobacteria, respectively to the bottom anoxic parts of the water body, thus, depriving them of favorable conditions and successfully controlling the blooms.

Although all the aforementioned methods are highly effective, there are various other biotic and abiotic factors that influence the growth of harmful algal blooms. Zuo et al. investigated the biotic factors that influenced the sustainability of the toxic cyanobacterial blooms in China’s Lake Taihu and revealed that the α-proteobacteria *Phenylobacterium* promoted the growth and dominance of toxic *Microcystis aeruginosa* strain [117]. Other abiotic factors include temperature, carbon dioxide (CO_2_) concentration and oxygen (O_2_) availability that influence the growth of toxic cyanobacterial blooms.

While the above-mentioned methods have been used for HAB mitigation efforts, no optimal method has been developed as yet. Furthermore, there is no effective treatment that is available for degrading microcystins in the natural environment. Complete removal of cyanotoxins from drinking water still remains a concern worldwide. As low doses of HAB toxins can exacerbate pre-existing comorbidities, some researchers have focused on using lactic acid bacteria such as *Lactobacilli* and *Bifidobacteria* as a pro-biotic to effectively degrade MCs. In a study done by Zhao et al., the researchers have shown these strains have free radical scavenging ability, as well as the ability to inhibit pro-oxidative enzymes and inhibit oxidative stress [118]. In a series of studies done by Nybom et al., the researchers demonstrated that the different strains of lactic acid bacteria can degrade MC-LR at optimum temperature and bacterial concentrations [119]. In another study by the same group, the authors have shown that a combination of lactic acid bacteria enhanced the degradation as compared to individual strains alone [120]. These authors also suggested a mechanism whereby the bacteria degraded MC-LR via extracellularly located cell-envelope proteinases [121,122].

Interestingly, MC degrading bacteria have even been reported from HAB-affected waters around the world, including Lake Erie [123,124]. Thees et al. have isolated and identified a select group of five MC degrading bacteria from Lake Erie that were found to degrade MC-LR to non-toxic forms and could potentially be used to remove MCs from drinking water Erie [123,124]. Additionally, other common bacterial strains belonging to the *Lactobacilli* and *Bifidobacter* species have demonstrated their ability to degrade MCs both in vitro and in vivo as discussed above [118,119]. The facility of these bacteria to rapidly degrade MCs suggests their potential to not only remove MC-LR from drinking water but also potentially to be used as a pro-biotic preventative therapy; however, further research to explore this area is required.

## 8. Summary

Cyanobacterial blooms are a persistent and growing problem that will require tremendous and concerted efforts to combat. While various efforts are being made in the way of the prevention of HABs, the unfortunate reality is that these blooms have occurred for years and are expected to continue to occur into the foreseeable future. Therefore, not only have humans and animals already been exposed for years, but they will continue to be exposed until the source is eliminated. As pointed out earlier, the current established guidelines for cyanotoxin exposure are based on studies performed in healthy animals; however, the potential chronic effects of HAB toxins such as MCs, especially in vulnerable populations such as those with pre-existing liver, gastrointestinal, or lung disease, deserve attention as there are limited data in this area. Additional research is required to define at-risk populations whose underlying comorbidities may confer increased susceptibility to HAB toxin exposure. A mechanistic understanding of chronic, low dose exposure to HAB toxins is needed so that appropriate preventative, diagnostic, and therapeutic strategies can be created in a targeted fashion. Apart from basic control measures such as nutrient limitation, as well as physical and chemical methods to limit the growth and spread of HABs, there is an immediate need to provide corrective measures by developing safe and efficient ways of HAB management in order to mitigate their detrimental effects to humans and the environment.

## Data Availability

All reported data are available via the corresponding authors upon request.
